# Integrative analysis of the inter-tumoral heterogeneity of triple-negative breast cancer

**DOI:** 10.1038/s41598-018-29992-5

**Published:** 2018-08-07

**Authors:** Alec M. Chiu, Mithun Mitra, Lari Boymoushakian, Hilary A. Coller

**Affiliations:** 1 Bioinformatics Interdepartmental Program, University of California, Los Angeles, USA; 2 Department of Molecular, Cell, and Developmental Biology, University of California, Los Angeles, USA; 3 Department of Biological Chemistry, David Geffen School of Medicine, University of California, Los Angeles, USA; 4 Department of Computer Science, University of California, Los Angeles, USA

## Abstract

Triple-negative breast cancers (TNBC) lack estrogen and progesterone receptors and HER2 amplification, and are resistant to therapies that target these receptors. Tumors from TNBC patients are heterogeneous based on genetic variations, tumor histology, and clinical outcomes. We used high throughput genomic data for TNBC patients (n = 137) from TCGA to characterize inter-tumor heterogeneity. Similarity network fusion (SNF)-based integrative clustering combining gene expression, miRNA expression, and copy number variation, revealed three distinct patient clusters. Integrating multiple types of data resulted in more distinct clusters than analyses with a single datatype. Whereas most TNBCs are classified by PAM50 as basal subtype, one of the clusters was enriched in the non-basal PAM50 subtypes, exhibited more aggressive clinical features and had a distinctive signature of oncogenic mutations, miRNAs and expressed genes. Our analyses provide a new classification scheme for TNBC based on multiple omics datasets and provide insight into molecular features that underlie TNBC heterogeneity.

## Introduction

Breast cancer is heterogeneous in nature, as breast tumors can arise from different cells of origin, and can present with distinct mutational signatures, biological and clinical phenotypes, and survival outcomes^1–3^. Breast tumors have been categorized clinically based on histological analyses of the levels of two hormonal receptors, estrogen (ER) and progesterone (PR), and human epidermal growth factor receptor 2 (HER2) amplification^4^. This classification system has been proven to have predictive power about a patient’s prognosis and is routinely used to make decisions about therapy. Perou *et al*. analyzed the inter-tumor heterogeneity in breast cancer using microarrays and developed a classification system that defined intrinsic subtypes (luminal A, luminal B, HER2-enriched, basal-like, and normal)^5^. New breast tumors can now be assigned to one of these classes based on the expression pattern of 50 informative genes termed PAM50 (ref.^5^). These breast cancer subtypes have different molecular characteristics and clinical outcomes^6^. Luminal A has the best clinical outcome. Comparing luminal A and luminal B tumors, luminal B tumors have higher expression of cell cycle and cell proliferation genes, lower expression of luminal genes such as PR, higher mutational burden and more copy number changes. HER2-enriched tumors have the highest mutational burden of all subtypes and display high expression levels of HER2-regulated and cell proliferation genes. Basal-like tumors highly express cytokeratins that are usually present in the basal cells of normal breast, and cell proliferation genes. Another intrinsic subtype called claudin-low was subsequently identified^7^. The claudin-low subtype is associated with signatures related to mammary stem cells and epithelial-to-mesenchymal transition. In a large-scale study (~500 breast cancer patients) by The Cancer Genome Atlas (TCGA), the authors sought to identify breast cancer subtypes based on a combination of genomic, mutational, and epigenetic signatures. This study revealed four groups that showed high concordance with the four intrinsic PAM50 subtypes^8^.

The majority of breast cancers (80–85%) are positive for hormonal receptors and/or HER2 amplification, and therefore respond to therapies that target these markers^6^. Triple negative breast cancer (TNBC) is clinically defined based on the absence of the estrogen and progesterone receptor, and HER2 overexpression. TNBC constitutes approximately 16% of breast cancer cases^9^. It is a particularly proliferative and aggressive subtype of breast cancer, associated with large size, high tumor grade, high mitotic rate, and metastasis^10^. Because of the lack of druggable targets, TNBC are treated with conventional chemotherapy, and only approximately 50% of patients respond^11^. The 5-year survival rate is only 14%^12^, much worse than for other breast cancer subtypes. TNBC are more prevalent in young women and in women of African or Hispanic descent^13^.

TNBC demonstrates genomic heterogeneity, as there is a substantial variation in mutational burden, copy number alterations, and genomic rearrangements across TNBC patients^14–16^. In contrast to other types of breast cancer in which several genes are mutated at high frequency, only two genes (TP53 and PIK3CA) are mutated in >10% of TNBC patients^16,17^. Thus, there are many different oncogenic drivers of TNBC. TNBC also demonstrates histological heterogeneity as nine different histologic subtypes have been identified based on immunohistochemistry (IHC), with invasive ductal carcinoma being the most prevalent type^15,16^.

Understanding the molecular basis of TNBC heterogeneity will be important for the development of precision therapy that targets specific molecular markers present in some TNBCs (genes, miRNAs etc.). Previous studies have defined subtypes of TNBC based on gene expression^18–21^. Most TNBCs are classified as basal-like subtype based on PAM50 gene expression signatures^19^. Further classification schemes focused more specifically on TNBC have also been developed. Lehmann *et al*.^18^ and Burstein *et al*.^20^ identified six and four TNBC subtypes, respectively, using microarray analysis. The six subtypes defined by Lehmann *et al*. have unique gene expression signatures and respond differently to chemotherapy. Some of the gene expression signatures associated with two (immunomodulatory and mesenchymal stem-like) of the six subtypes likely reflect the presence of cells from the tumor microenvironment, rather than the cancer cells themselves^19,21^. To address this, Lehmann *et al*. recently published a revised classification containing four TNBC subtypes (basal-like 1 (BL1), basal-like 2 (BL2), mesenchymal (M), and luminal androgen receptor (LAR)) using RNA-seq data from TCGA^21^. The survival outcome of patients with the BL1 subtype was found to be significantly better than the survival for patients from the other three subtypes combined.

Inter-tumor heterogeneity for breast cancers, including TNBC, has been measured mainly by clustering tumors based on gene expression data from microarray or RNA sequencing methods. The availability of data from other sources (miRNA, DNA methylation, CNVs, protein arrays) has provided new avenues to understand tumor heterogeneity. Recently, three TNBC subtypes based on DNA methylation sequencing have been proposed and shown to have different clinical courses^22^. Integrative clustering methods^23,24^ combining gene expression data with other data types such miRNAs and CNVs have been performed to identify novel subtypes for breast^25,26^ and other tumors^27^. Integrative approaches have been shown to be more robust in comparison to methods using biomarkers of one type only^23^. To date, methods that integrate different datatypes have not been applied to understand TNBC heterogeneity.

Given the clinical importance of TNBC and the clear need for classification systems that capture the inter-tumor heterogeneity of TNBC in a comprehensive manner, we performed integrative clustering combining information from multiple types of omics data (gene expression, miRNA expression, and CNV) collected from TNBC tumors. This led to the identification of three TNBC clusters with distinct molecular and clinical features. This new classification scheme may have implications for the treatment of TNBC.

## Results

### Selection of TNBC patients

Out of 1098 breast invasive carcinomas in the TCGA database, 180 have been classified as triple-negative by Lehmann *et al*. based on low expression levels of ER, PR, and HER2^21^. In addition to the cancer cells, a tumor sample may contain cells from the tumor microenvironment (TME), such as fibroblasts, endothelial cells, and immune cells. The presence of these cells may confound the signals originating from cancer cells. Sequencing data provided by TCGA were collected from only those cancer tissues with >60% cancer cells (>60% tumor purity) as determined by counting the tumor nuclei^28^. Recently, more refined computational methods using sequencing data (gene expression, methylation, and CNV) have been used to estimate tumor purity by taking into account the contribution of non-cancer cells^29^. Aran *et al*. provided consensus purity estimations (CPE) based on a combination of four methods—gene expression of non-cancer cells, somatic CNVs, immune-related methylation pattern, and immunohistochemical analysis. In an independent analysis, tumor purity scores (from Clonal Heterogeneity Analysis Tool (CHAT)) for the same tumors were determined based on an analysis of copy number alterations^30,31^. For this study, we only included those TNBC primary tumors for which the tumor purity scores were estimated to be >60% by either Aran *et al*. or Li *et al*. (Supplementary Table S1). One hundred thirty-seven out of 180 TNBC tumors met these criteria. About 39% of these patients were 50 years of age or younger (Table 1) and most of these patients (76%) had tumors that were classified as basal-like PAM50 subtype.

**Table 1:** Clinical characteristics of 137 TNBC patients.

Feature	Number of Patients (% total)
Age	25–50 years: 54 (39.3%)
	50–75 years: 73 (53.3%)
	>75 years: 10 (7.3%)
Ethnicity	Caucasian: 83 (60.6%)
	African descent: 40 (29.2%)
	Asian: 7 (5.1%)
	Not available: 7 (5.1%)
PAM50 subtypes	Luminal A: 16 (11.7%)
	Luminal B: 2 (1.5%)
	HER2-enriched: 6 (4.4%)
	Basal-like: 104 (75.9%)
	Normal: 9 (6.6%)
Lehmann subtypes	Basal like 1 (BL1): 44 (32.1%)
	Basal like 2 (BL2): 23 (16.8%)
	Luminal androgen receptor (LAR): 26 (19%)
	Mesenchymal (M): 43 (31.4%)
	Unclassified (UNC): 1 (0.7%)

### Single-data clustering to determine TNBC heterogeneity

**Figure 1:**
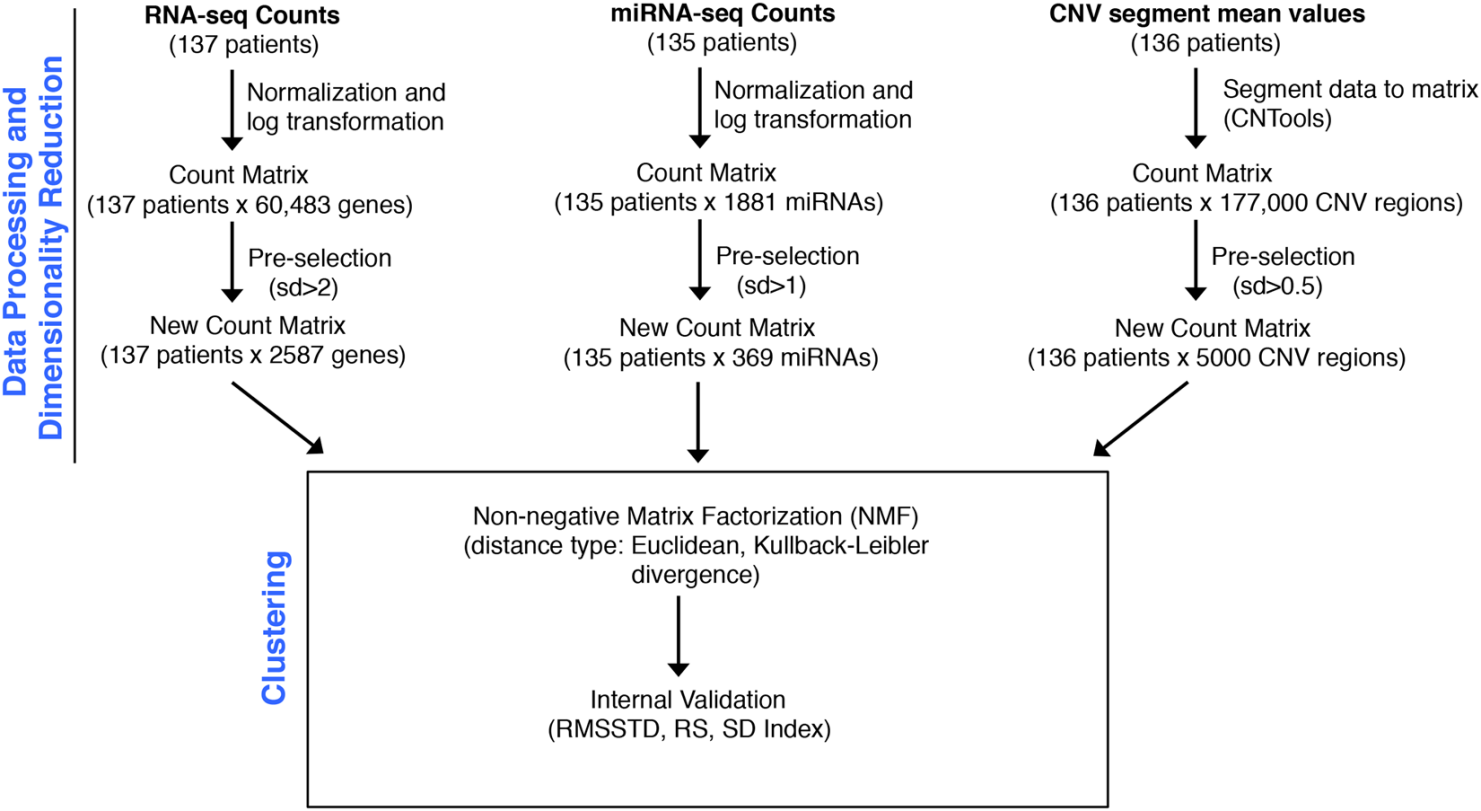
Workflow for single-data clustering of TNBC patients. Data for gene expression (RNA-seq counts), miRNA expression (miRNA-seq counts) and copy number variations (CNVs) were obtained from the Genomics Data Commons portal (https://portal.gdc.cancer.gov/) for all patients with >60% tumor purity (percent of cancer cells in a tumor mass) based on the scores provided by Aran *et al*.^55^ and Li *et al*.^30^. Two patients were excluded from miRNA-based clustering and one patient was excluded from CNV-based clustering due to unavailability of data. All the data were processed to generate a matrix with all of the data for each patient. This matrix was reduced in dimensions by including only the genes, miRNAs and CNVs that were most variable among the patients (referred to as the “pre-selection step”). This pre-selection step for dimensionality reduction (count matrix → New count matrix) was performed based on a defined standard deviation (sd) cutoff (sd >2 for genes; sd >1 for miRNAs; sd >0.5 for CNVs) to select the most variable genes, miRNAs, and CNV regions. Clustering for all three data types was performed with a clustering method (non-negative matrix factorization (NMF)) designed to find orthogonal matrices similar to finding multiplicative factors of a number. The clusters that resulted from NMF for each of the data types (gene, miRNA, or CNV) were tested with three different methods, root-mean-square standard deviation (RMSSTD), r-squared (RS), and SD index, that allow for a determination of compactness (RMSSTD, RS) of individual patient clusters and the separation (SD index) of the patients clusters^34^.

In order to assess inter-patient heterogeneity among the TNBC samples, we performed consensus clustering (combining clustering information from different runs into one cluster)^32^ of patients using non-negative matrix factorization (NMF)^33^. Consensus clustering was performed separately using gene expression (RNA-seq), miRNA expression (miRNA-seq), and copy number variants (CNV) (SNP 6.0 array) data provided by TCGA (Fig. 1, see Methods). Consensus clustering was performed on the subset of genes, miRNAs, and CNVs with the largest variation across the patients (pre-selection step, Fig. 1).

Gene expression-based NMF clustering was performed on 137 patients using two different distance metrics: Kullback-Leibler and Euclidean. To test the quality of the clusters, three different validation metrics^34^ were applied that analyzed the degree of cluster compactness (RMSSTD, r-squared) and cluster separation (SD validity index). Out of two distance metrics, the Euclidean metric performed the best based on top scores in all the three validation tests (Supplementary Table S2). For Euclidean metric-based clustering (NMF-gene), the optimal number of clusters was determined to be four (see Methods) (Supplementary Fig. S1). We also performed single-data clustering using miRNA expression and CNV data obtained from TCGA (Fig. 1). For both of these data types, the Euclidean distance metric again provided the best solution (Supplementary Table S2). Four and three cluster solutions were considered optimal for miRNA-based (NMF-miRNA) and CNV-based (NMF-CNV) clustering, respectively, using the Euclidean distance metric (Supplementary Fig. S1).

**Figure 2:**
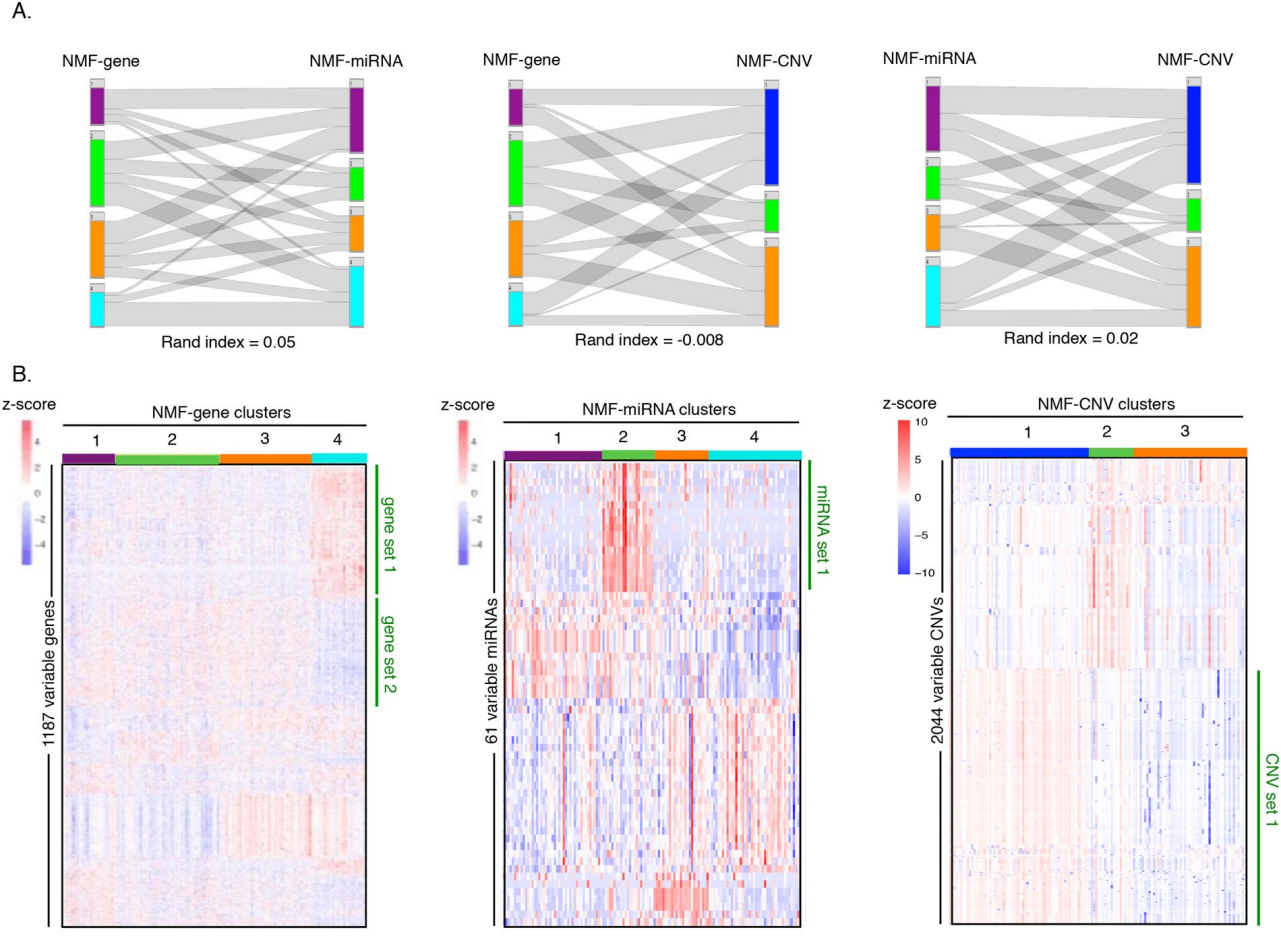
Relationship between single-data clusters. (**A**) Comparison of clusters generated by non-negative matrix factorization (NMF) method for different data types. The clusters (vertical bars of different colors) generated by gene expression data (NMF-gene, total four clusters) are compared with the clusters generated from miRNA expression data (NMF-miRNA, total four clusters) on the left. The assignment of individual tumors in the two schemes is shown with gray lines. A comparison of the clusters generated with gene expression data (NMF-gene) and the clusters generated with CNV data (NMF-CNV, total three clusters) are shown in the middle. The clusters generated with miRNA data (NMF-miRNA) were compared with clusters generated with CNV data (NMF-CNV). The plots were prepared using the StratomeX package^70^. The adjusted rand index^35^ values, a measure of the similarity between the clusters produced with the different data types that ranges from 0 to 1 with 1 being perfect correlation, are also shown. (**B**) The heat map on the left shows the 1187 most variable genes among the four NMF-gene clusters (same color scheme as A) with each row representing a gene. As shown on the z-score scale, red indicates high expression for a particular gene in a particular tumor compared with the other tumors, while blue indicates low expression of a particular gene in a particular tumor compared with other tumors. The heat map in the middle shows the expression level of the 61 most variable miRNAs between the four tumor clusters of NMF-miRNA. The heat map on the right shows the 2044 most variable CNVs across the three tumor clusters developed from CNV data (NMF-CNV). The order of the genes was determined by a hierarchical clustering algorithm. A Kruskall-Wallis statistical test was used to identify the most variable genes, miRNAs, and CNVs that were included in the heat maps, using a false discovery rate (FDR) <0.10.

Comparison of the best NMF clustering solutions obtained from three data types (NMF-gene, NMF-miRNA, and NMF-CNV) did not show much agreement as indicated by very low (NMF-gene/NMF-miRNA and NMF-miRNA/NMF-CNV) and negative (NMF-gene/NMF-CNV) adjusted rand index scores^35^ (a metric of the similarity between two clustering solutions) (Fig. 2A). To gain further insight into these single-data NMF clusters, we determined the signature genes, miRNAs, and CNVs that displayed the largest variation between the clusters for NMF-gene (1187 genes), NMF-miRNA (61 miRNAs), and NMF-CNV (2044 CNVs), respectively (see Methods). This identified gene sets 1 and 2, miRNA set 1, and CNV set 1 that showed prominent variation between the clusters (Fig. 2B). Thus, different data-types captured different information about the heterogeneity in TNBC tumors.

### Comparison of single-data clusters with PAM50 and Lehmann subtypes

For each of the NMF-miRNA and NMF-CNV clusters, more than half of the tumors were classified as basal-like PAM50 subtype (Supplementary Fig. S2). The fraction of basal-like tumors in these clusters ranged from 57–84% for miRNA-based clusters and 70–81% for CNV-based clusters. For NMF-gene clustering, the fraction of basal-like tumors in three out of four clusters was found to be >65% (Supplementary Fig. S2). These results agree with previous studies showing TNBC tumors to be predominantly of basal-like subtype^19^. The clustering indicates heterogeneity within the basal-like subtype as these tumors did not cluster together, but rather were split into separate clusters. One exception among single-data clusters was cluster 4 of the NMF-gene classification (Supplementary Fig. S2). The tumors in this cluster were composed primarily of luminal A (38%) and HER2-enriched (25%) subtypes. Among the NMF clusters, the NMF-gene clusters correlated best with Lehmann subtypes, which were developed based on gene expression data (Supplementary Fig. S2). The majority of patient tumors from cluster 1 were M subtype; the majority of patients in cluster 3 were basal-like; and the majority of the patients in cluster 4 were LAR.

### Integrative clustering of TNBC patients

Integrative clustering methods that combine different data types are based on more “information content” than methods that rely on a single data clustering^23^. They consequently have the potential to provide a comprehensive view of inter-tumor heterogeneity. The low concordance among the single-data clusters from the three data types (Fig. 2A) suggests that the different data types capture the variation among the tumors in different ways. We reasoned that integrative clustering might be better suited for TNBC classification because it would combine information from the three data types. The similarity network fusion (SNF) method^36^ was used to integrate gene, miRNA, and CNV data from 134 TNBC patients. Each of the three data types was first used to construct individual patient networks. These networks were then combined to generate a single fused network. Spectral clustering was then performed on this fused network to reveal patient clusters or subtypes. In contrast to single-data clustering (Fig. 1), no pre-selection of genes, miRNAs, or CNVs was required for SNF analysis, thus removing any bias due to a pre-selection step.

**Figure 3:**
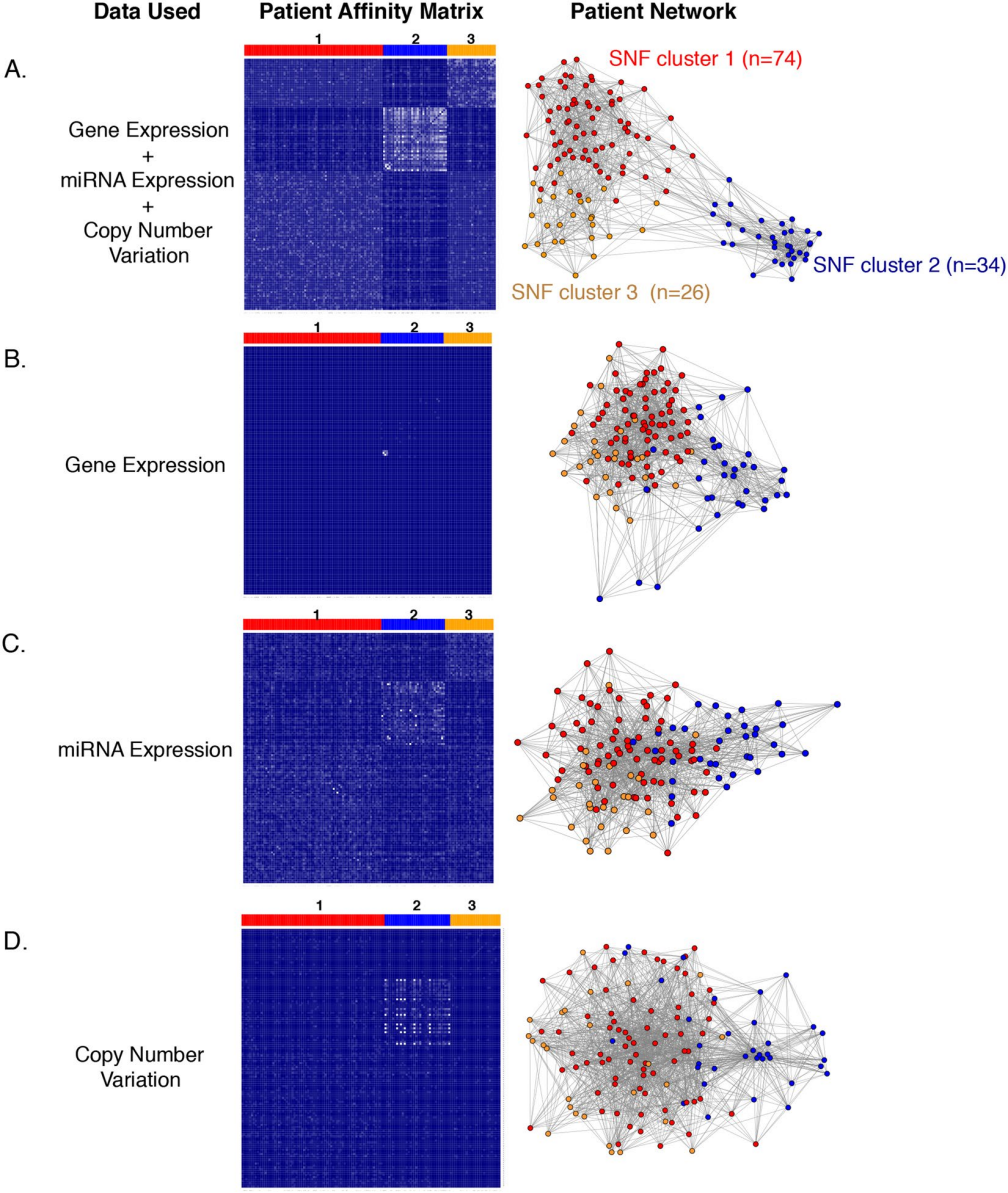
Similarity network fusion (SNF)-based integrative clustering of patient tumors. (**A**) Patient tumors were clustered based on a combination of gene expression, miRNA expression and copy number variants. Heatmap (left) for the patient-to-patient similarity matrix generated using the SNF method by combining gene expression, miRNA expression, and CNV data. The white and blue regions represent high and low similarities between patients, respectively. The patients in the heatmap are grouped according to the clusters (cluster 1 in red, cluster 2 in blue, and cluster 3 in orange) obtained by spectral clustering of the similarity matrix. On the right, the patients are represented as a network with patients as nodes (dots) connected by edges (lines). Long edges connect patients with low similarity, while short edges connect patients with high similarity. (**B**–**D**) Heatmaps (patient-to-patient similarity matrices) are shown on the left and networks are shown on the right for clusters developed based on a single-data type: gene expression data (**B**) miRNA data (**C**) or copy number variant data (**D**). For both heatmaps and networks, the patients are clustered and color-coded based on the clusters shown in (**A**).

SNF-based clustering produced three clusters (SNF clusters 1–3) upon combining gene, miRNA, and CNV data (Fig. 3A). The three SNF clusters were less distinct when considering patient networks based on a single data type (Fig. 3B,C and E). Coverage scores (ratio of intra-cluster edges to total edges)^37^ for SNF clusters showed higher coverage for the fused patient network constructed using the combination of three data types (gene + miRNA + CNV, 85%) compared to those generated by single-data types (gene only, 69%; miRNA only, 67%; and CNV only, 58%). The higher fraction of edges connecting the nodes within the clusters for the fused network indicates better separation (lower fraction of inter-cluster edges) compared to single-data types. The separations among SNF clusters 1–3 were maintained when DNA methylation data was incorporated with gene, miRNA, and CNV data (see Methods) to generate the fused network (Supplementary Fig. S3).

### Comparison of SNF clusters with single-data clusters, PAM50, and Lehmann classifications

The clusters generated by an integration of the three datatypes did not correspond to the clusters generated with any single datatype using NMF clustering methods. SNF Cluster 2 had the most overlap with NMF-gene cluster 4 (71%), NMF-miRNA cluster 4 (68%), and NMF-CNV cluster 1 (71%) (Supplementary Fig. S4). Most of the patient tumors in SNF cluster 3 were assigned to NMF-gene cluster 1 (63%) and NMF-miRNA cluster 1 (63%). There was no major overlap (>50%) of SNF cluster 1 with any of the single-data clusters.

**Figure 4:**
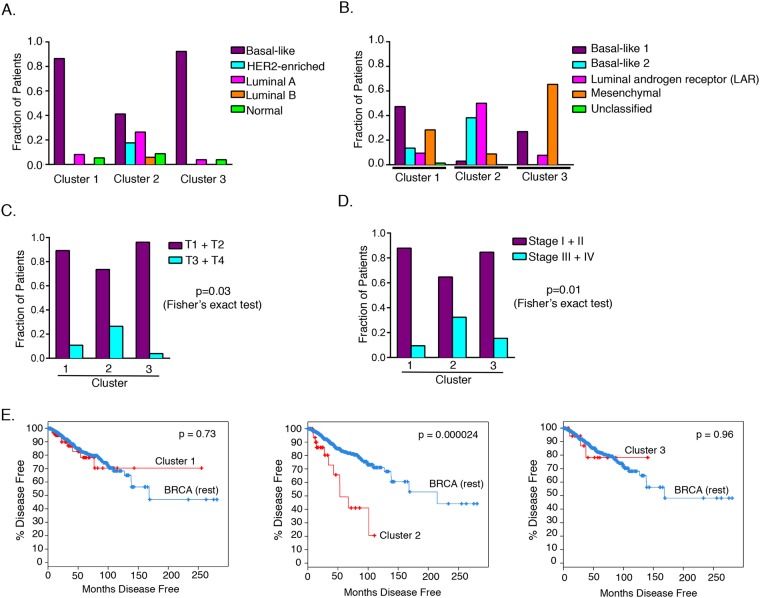
Clinical properties of integrative clusters. (**A**) Classification of the three clusters obtained by integrative analysis (similarity network fusion method) of genes, miRNAs, and CNVs based on gene expression-based PAM50 breast cancer subtypes^5^. (**B**) Classification of the three integrative clusters based on gene expression-based TNBC subtypes developed by Lehmann *et al*.^21^. For each SNF cluster, the fraction of patients belonging to each of the five PAM50 (**A**) and Lehmann (**B**) subtypes are shown. (**C**,**D**) Patients in each of the three integrative clusters are classified based on two clinical measures: pathologic T (**C**) and pathologic stage (**D**). Pathologic T is based on the size of tumor and extent of tumor growth into nearby tissues, while pathologic stage is based on the combined analysis of tumor size, lymph node metastasis, and metastasis to distant organs. (**E**) Comparison of disease-free survival of an integrative cluster with the TCGA breast cancer patient cohort (breast invasive carcinomas, n = 1098) excluding the patients associated with this integrative cluster.

The SNF clusters that we generated did not align with the PAM50 intrinsic subtypes defined for all breast cancers (Fig. 4A) or the subtypes defined by Lehmann *et al*. specifically for TNBC (Fig. 4B). Of the three SNF clusters, the fraction of tumors with a basal-like subtype classification based on PAM50 was least for cluster 2 (41%) compared to clusters 1 (86%) and 3 (92%) (Fig. 4A). Cluster 2 had higher fractions of HER2 (18%) and luminal (32%) PAM50 subtypes compared to clusters 1 (0% HER2 and 4% luminal) and 3 (0% HER2 and 8% luminal). When compared with the Lehmann subtypes (Fig. 4B), clusters 1 and 3 were mainly composed of basal-like 1 (47%) and mesenchymal subtypes (65%), respectively, while basal-like 2 (38%) and luminal androgen receptor subtypes (50%) represented the majority of the tumors in cluster 2. The SNF clusters we generated did share a similarity to NMF-gene clusters (Supplementary Fig. S2) and Lehmann subtypes^21^ in that they all contained a cluster (cluster 4 of NMF-gene and LAR subtype by Lehmann *et al*.) that was enriched in HER2 and luminal PAM50 subtypes, with the rest of the clusters containing a high fraction of tumors with a basal-like subtype.

**Figure 5:**
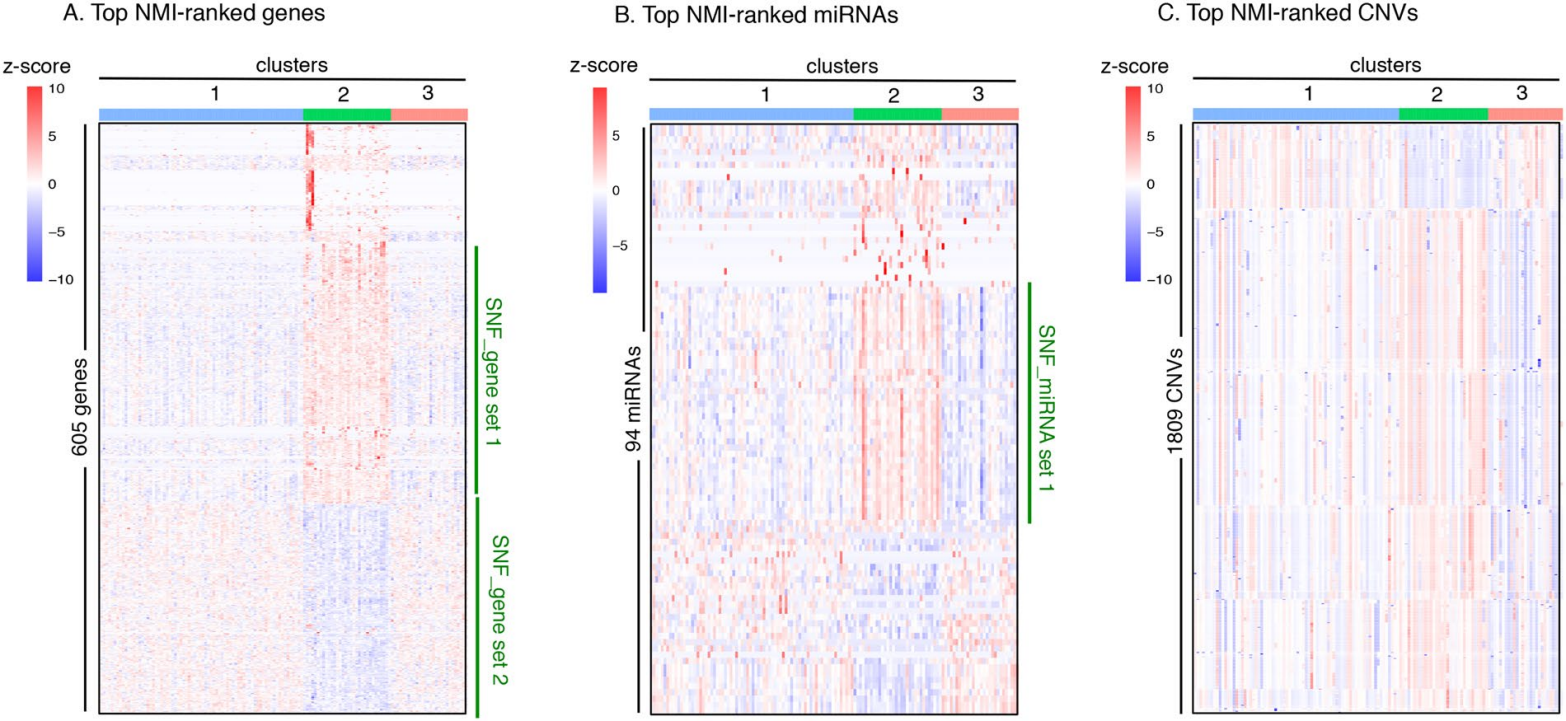
Heatmaps of top scoring features across the integrative clusters. The features (genes, miRNAs, and CNVs) are scored based on their normalized mutual information (NMI) values obtained by similarity network fusion (SNF)-based integrative clustering of genes, miRNAs, and CNVs. Higher NMI scores for a feature (gene, miRNA, or CNV) indicate that the feature was more important for the fused network generated by SNF. Heatmaps are shown for the genes (**A**) miRNAs (**B**) and CNVs (**C**) with the top NMI scores. To generate the heatmaps, the top 1% of genes (605), the top 5% of miRNAs (94), and the top 1% of CNVs (1809) were selected and the variation of their levels across the integrative clusters was depicted. Higher levels are shown in red and lower levels are presented in blue.

### Clinical properties of SNF clusters

SNF cluster 2 had a greater fraction of patients (26%) with larger tumor size (tumor score of T3 and T4 in TNM staging system), compared to clusters 1 (11%) or 3 (4%) (p = 0.03, Fisher’s exact test) (Fig. 4C). Higher T numbers (T3 and T4) indicate larger tumor size and/or greater extent of tumor growth into nearby tissues^38^. Thirty two percent of cluster 2 patients had tumors of higher pathologic stage (stages III and IV). In comparison, only 9% and 15% of cluster 1 and cluster 3 patient tumors, respectively, were of stage III and stage IV (p = 0.01, Fisher’s exact test) (Fig. 4D). In addition to tumor size, pathologic stage is also dictated by regional lymph node and distant organ metastasis^38^. Disease-free survival analysis revealed that cluster 2 TNBC patients (n = 34) had poorer disease-free survival (p = 0.000024) compared to the remaining breast cancer patient cohort from TCGA (breast invasive carcinomas, n = 1098) (Fig. 4E). In contrast, no significant difference was found between disease-free survival for clusters 1 (n = 74) or 3 (n = 26) compared with the remaining breast cancer patient cohort.

### Differentially expressed genes and miRNAs between SNF clusters

In order to understand the biological basis for the segregation of the TNBC tumors into the clusters generated with integrated SNF clustering, we ranked the features (genes, miRNAs, and CNVs) based on their normalized mutual information (NMI) scores^36^ (see Methods section of Cavalli *et al*.^27^). These scores (Supplementary Table S3) provide a measure of the importance of different features for the construction of the fused network obtained by SNF. Analysis of all the features with the top-ranked NMI scores (top 1% genes, top 5% miRNAs, and top 5% CNVs) across the three SNF clusters revealed a subset of genes and miRNAs (gene set 1, gene set 2, and miRNA set 1) that were differentially expressed in cluster 2 compared to clusters 1 and 3 (Fig. 5). Thus, the top-ranked NMI features contributed more toward separation of cluster 2 from clusters 1 and 3. The 605 genes with the top 1% NMI scores (Fig. 5A) were enriched for genes related to transcriptional regulation, mesenchymal cell differentiation, embryo development and morphogenesis. The gene targets for the top 5% NMI-ranked miRNAs (94 miRNAs) (Fig. 5B) were involved in positive regulation of cellular and metabolic processes, development, and cell proliferation. The genes affected by the top 5% NMI-ranked CNVs (1809 CNVs) (Fig. 5C) participate in amino acid metabolism and phosphatidylcholine 1-acylhydrolase activity. These CNVs are mainly present on chromosomes 1, 5, and 14 (Supplementary Fig. S5). Out of 1809 CNVs, about 722 CNVs were not closely associated with any genes.

**Figure 6:**
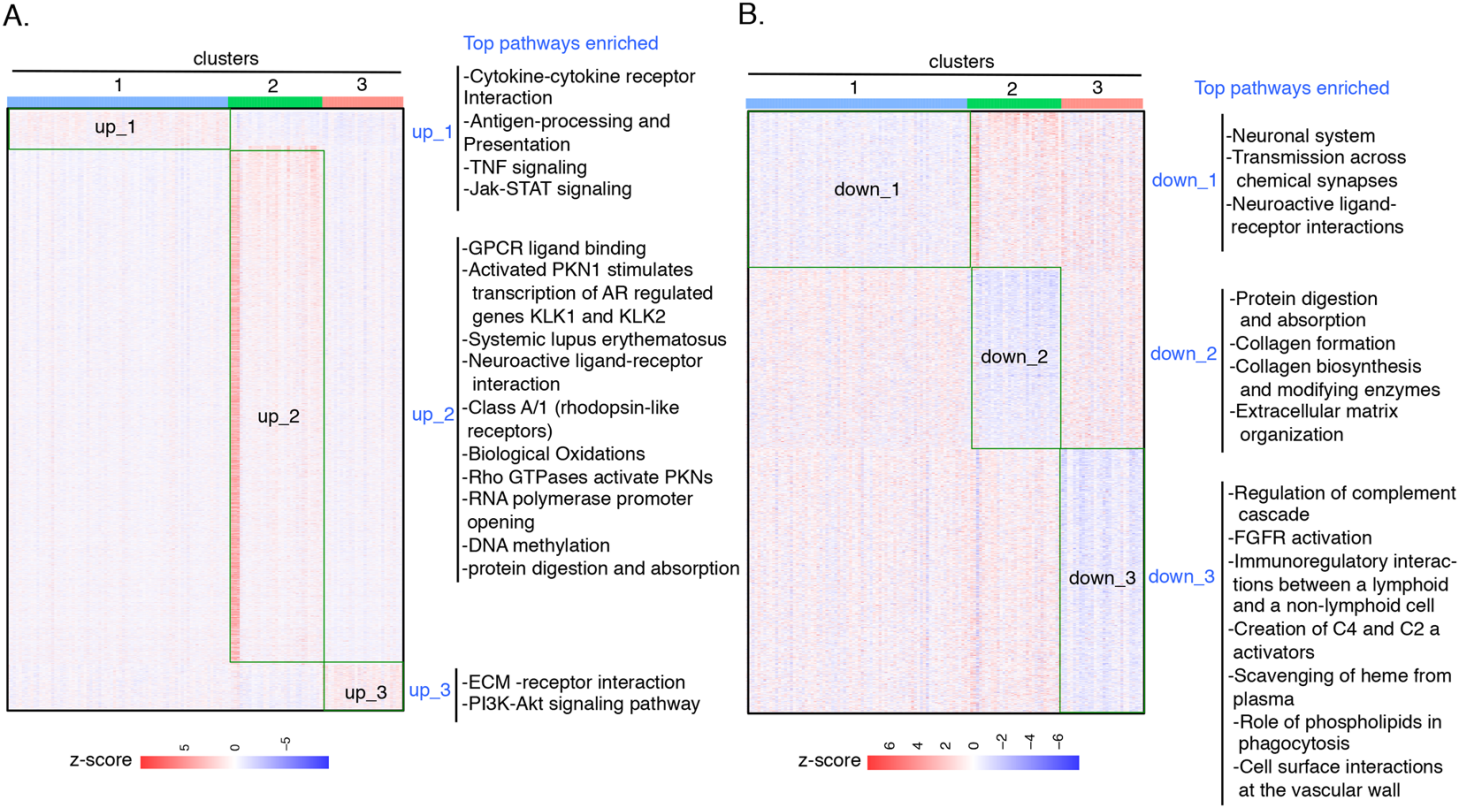
Top cellular pathways related to integrative clusters. (**A**) Heat map showing the gene sets (boxed regions), up_1, up_2, and up_3 that are specifically upregulated in clusters 1, 2, and 3 respectively. Each row represents a gene and each column is a tumor. Higher scores shown in red indicate higher levels of gene expression in a specific tumor and lower scores shown in blue indicate lower expression levels in a specific tumor. (**B**) Heat map showing the gene sets (boxed regions) down_1, down_2, and down_3 that are specifically downregulated in clusters 1, 2, and 3 respectively. For both (**A** and **B**) the most significant pathways enriched in upregulated and downregulated gene sets are shown. The biological pathway analysis was performed using the g:Cocoa module of g:Profiler^63^.

Analysis of the genes with the top NMI scores revealed differences between cluster 2 and clusters 1 and 3 (Fig. 5, gene sets 1 and 2 and miRNA set 1). In order to find signature genes specific to clusters 1, 2, and 3, we performed differential gene expression analysis for three cluster pairs (1 vs. 2, 2 vs. 3, and 1 vs. 3) and then extracted the genes that are specifically upregulated (up_1, up_2, and up_3) or downregulated (down_1, down_2, and down_3) in each of the three clusters (Supplementary Table S4 and Fig. 6). Of the top 100 NMI-scored genes, 68 were specifically upregulated (46 genes) or downregulated (22 genes) in cluster 2. This supports the importance of cluster 2-specific genes in determining the pattern of the fused patient network. The gene ontology (Supplementary Table S4) and pathway analysis (Fig. 6) suggests that different cellular pathways are activated in clusters 1, 2, and 3. For instance, the androgen receptor (AR) was specifically upregulated in cluster 2 (up_2, Supplementary Table S4). AR-dependent signaling is activated in tumors classified by Lehmann as LAR subtype^39^ and 50% of cluster 2 tumors are classified as LAR subtype (Fig. 6B). Pathway terms enriched among the genes induced in cluster 2 include “Rho GTPases activate PKNs” and “activated PKN1 stimulates transcription of AR regulated genes KLK1 and KLK2”, thus identifying a signaling pathway associated with androgen receptor expression upregulated in cluster 2. The expression of cluster 2 -specific genes (up_2 and down_2) was similar across the PAM50 and Lehmann subtypes (Supplementary Fig. S6).

The miRNAs specifically upregulated and downregulated in each of the three SNF clusters (Supplementary Table S5) were obtained using the same strategy used to extract cluster-specific genes. Fifty-two of the top 100 NMI-ranked miRNAs (38 upregulated and 14 downregulated) were unique to cluster 2. The oncogenic miRNA *miR-10b* was specifically upregulated in cluster 2. Overexpression of *miR-10b* in non-metastatic breast cancer cells has been shown to induce cancer cell invasion and metastasis, and expression of *miR-10b* in primary breast tumors is associated with metastasis^40,41^. Two tumor suppressor miRNAs^41,42^, *miR-17* and *miR-206*, were downregulated in cluster 2. *miR-17*-*5p* targets amplified in breast cancer (AIB1), which in turn promotes cell proliferation^43^. miR-206 is downregulated in many cancers and has been shown to target K-Ras, annexin a2, and cell cycle genes^44,45^. Oncogenic miRNA *miR-9* (produced from the *mir-9-3* locus) was upregulated in cluster 3^46^. *miR-9* targets E-cadherin and thereby promotes cell migration and invasion. As with genes, the expression of cluster 2-specific miRNAs (up_2 and down_2) did not vary depending on PAM50 and Lehmann subtypes (Supplementary Fig. S6).

### Structural variations and mutational profile of SNF clusters

Genomic alterations such as single-base mutations and CNVs (covering regions >1 kilobase) are important drivers of carcinogenesis and can maintain the fitness of cancer cells^47,48^. These alterations are a potential source of inter-tumor heterogeneity. We determined the extent of gene amplifications due to CNVs in the tumors that constitute the three SNF clusters (Supplementary Table S6). Clusters 1 and 3 had 126 genes and 168 genes with amplification frequencies above 10%, respectively. In contrast, only 28 genes were altered with >10% frequency in cluster 2.

In addition to CNVs, activating and inactivating mutations can also serve as oncogenic drivers. Mutational analysis showed that tumor protein p53 (TP53) and titin (TTN) were commonly mutated in tumors in all three clusters with frequencies ranging from 63–78% and 23–27%, respectively (Supplementary Table S6). Phosphatidylinositol-4,5-bisphosphate 3-kinase catalytic subunit alpha (PIK3CA) was mutated at higher frequency in cluster 2 (23%) compared to clusters 1 (3%) and 3 (8%). Cluster 2 tumors contain a higher frequency of AR-positive tumors, and PIK3CA is frequently mutated in AR-positive tumors^39,49^.

### Predictive modeling and biological properties of predictors

**Figure 7:**
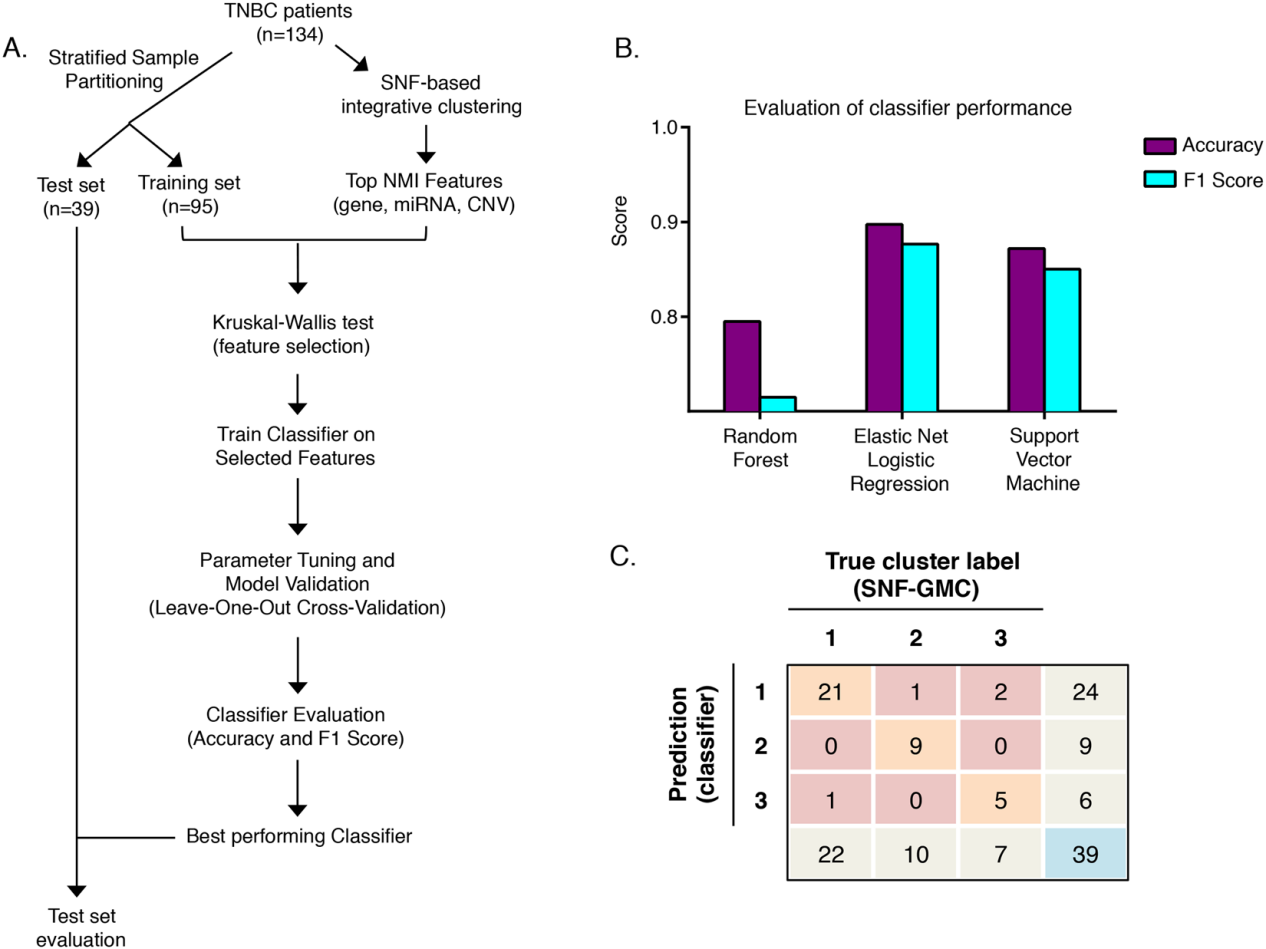
Classification algorithm to best predict the cluster assignment of a TNBC patient. (**A**) Workflow showing the steps involved in predictive modeling using the carat R package^69^ to find the best classification algorithm (classifier) based on the training set to predict cluster assignment (integrative cluster 1, 2 or 3) of a TNBC patient in the test group. The true cluster assignments of the patients in the test group are known based on the results of the integrative clustering. (**B**) The F1 score, which measures both precision and recall, and the accuracy are provided for three different classifiers tested: random forest, elastic net logistic regression, and support vector machine. (**C**) Matrix showing the performance of the elastic net logistic regression classifier on the test set. Rows represent the number of tumors predicted to be in each cluster by the classifier and the columns represent the known cluster for each of the tumors based on integrative clustering results. The entries in the diagonal (orange) contain tumors that are correctly predicted by the classifier.

Because the three SNF clusters have different biological and clinical properties, the ability to assign a new tumor to one of the three existing clusters could be beneficial for patients with TNBC. We sought to develop a classifier (see Methods) that would assign a new TNBC patient to one of the SNF clusters based on the patient’s mRNA, miRNA, and CNV data. The workflow for training and testing the classifier is shown in Fig. 7A. We divided the 134 patients used for SNF clustering into a training set (n = 95) for developing the classifier and a test set (n = 39) for classifier testing. We trained three different classifiers (random forest, elastic net logistic regression, and support vector machine) and evaluated their performances. The elastic net logistic regression had the highest accuracy (90%) and F1 score metrics (see Methods) (Fig. 7B and Supplementary Table S7). The accuracy for random forest and support vector classifiers were 79% and 87%, respectively. When applied to a test data set not used for training, the elastic net logistic regression classifer was able to correctly predict 35 out of 39 patient labels (clusters 1–3) indicating an accuracy of 90% (Fig. 7C). This classifier (see Methods) could be used to identify the cluster label of TNBC patients if gene, miRNA, and CNV data were available.

## Discussion

The breast epithelial layer consists of an inner layer of luminal cells and an outer layer of basal or myoepithelial cells^50^. Gene expression-based PAM50 intrinsic classification of breast cancer^5^ includes basal-like and luminal A/B subtypes, and two models have been proposed to explain the cellular origin of these tumor subtypes^50^. In the cell of origin model, luminal tumors originate from committed late progenitors of luminal cells, while basal tumors originate from early progenitors of luminal cells. The alternative genetic mutation model postulates that luminal and basal-like tumors result from different genetic insults acting upon similar populations of luminal progenitors. With this model, TNBC tumors result from additional changes that lead to a loss of expression of ER and PR in tumors without amplification of HER2. Much less is known about the origin of HER2-enriched PAM50 subtype. The tumors in this subtype are heterogeneous with respect to the expression of hormonal receptors (ER and PR) and only 70% of these tumors are classified as HER2+, suggesting a different cell of origin of these tumors^50^.

Using an integrative approach to clustering, we discovered that we could classify TNBC tumors into three distinct tumor clusters using a combination of gene, miRNA, and CNV data. Comparison of our integrative clusters with the PAM50 subtypes revealed that integrative clusters contained a mixture of tumors from multiple PAM50 subtypes. Cluster 1 and cluster 3 had a similar composition of tumors based on PAM50 classification. Tumors from both clusters were 80–90% basal-like, which is the subtype often associated with triple negative breast cancer, and contained a small amount of luminal A (4–8%), with normal making up the rest (Fig. 4A). In contrast, cluster 2 had the worst clinical features and disease outcome out of all the three integrative clusters, and the lowest proportion of patients identified as PAM50 basal subtype (41%). This cluster contained the highest proportion of HER2-enriched (18%) and luminal A (26%), with smaller contributions from luminal B and normal (Fig. 4A). Thus, by interrogating different types of features (genes, miRNAs, and CNVs) and interactions among them, our clustering resulted in distinct clusters from those produced by the PAM50 gene expression-only approach. We interpret the relationship between our integrative clusters and PAM50 gene expression as supporting a model in which there are two quite distinct pathways to TNBC, one involving mostly basal cells and leading largely to tumors in integrative clusters 1 and 3, and another that can result from luminal or HER2-enriched cells, that we believe leads to tumors in integrative cluster 2. These two pathways may reflect a different cell of origin or differences in the most critical oncogenic drivers.

We also compared the three clusters generated by our integrative analysis with the four TNBC subtypes of the gene expression-based Lehmann classification^21^. Each of the integrative clusters contains tumors that had been assigned to multiple different Lehmann subtypes (Fig. 4B). Integrative cluster 1 includes more basal-like 1 (47%) than the other subtypes, with mesenchymal (28%) the second most frequent. Integrative cluster 2, the cluster with poor clinical properties, is enriched in luminal androgen receptor (50%) and basal-like 2 (38%) Lehmann subtypes. The basal-like 1 Lehmann subtype, which has the best survival outcome compared to the rest of the subtypes combined^21^, is least represented in integrative cluster 2 (3%), suggesting agreement of integrative clusters with Lehmann subtypes in terms of clinical characteristics of the TNBCs. Cluster 3 of the integrative clustering was most strongly enriched (>60%) in the mesenchymal Lehmann subtype, with basal-like 1 (27%) the next most abundant. Comparing all of the classifications, we observe that three out of four Lehmann subtypes (BL1, BL2, and M) mostly consisted of TNBCs that were assigned as basal-like PAM50 subtype, while the majority of LAR tumors were HER2-enriched and luminal PAM50 types. Therefore, clusters 1 and 3 were similar in being mostly comprised of tumors classified as basal-like by PAM50 and as either basal-like 1 or mesenchymal by Lehmann. Cluster 2 was enriched for basal-like 2 and luminal androgen receptor by Lehmann classification, making it distinct from clusters 1 and 3, a finding also supported by its low levels of basal-like tumors by PAM50. By generating cluster 2, our integrative clustering found similarities between tumors called basal, HER2-enriched or luminal A by PAM50, or basal-like 2, luminal androgen receptor or mesenchymal by Lehmann classification, that were not detected by previous clustering approaches. Our findings also suggest that there are global distinctions identified by the features we considered that lead to two distinct and separable groups (cluster 1 and cluster 3) among tumors that are assigned basal-like by PAM50 and basal-like 1 or mesenchymal by Lehmann.

Each integrative cluster had a distinct profile with regard to genes, microRNAs and CNVs. Cluster 2 was the most distinct and its gene expression signature included genes in the RhoA-PKN1-androgen receptor pathway. PIKC3 was more frequently mutated in the tumors in this cluster, while copy number variants and gene amplification events were relatively rare. Tumors in this cluster also contained higher levels of the oncogenic microRNA *miR-10b*. Clusters 1 and 3 were similar to each other in terms of being mostly basal PAM50-subtype, similar disease-free progression and tumor size, similar numbers of copy number variants, and a similar set of oncogenic mutations with frequent mutations in p53 and titin. Tumors in clusters 1 and 3 were most strongly differentiated by downregulation of genes associated with the nervous system and synaptic transmission in the tumors in cluster 1 and downregulation of genes associated with the complement pathway and the humoral immune response in cluster 3. The variable expression of neuronal genes in TNBC clusters 1–3 is surprising, but neuronal-specific markers have been reported to be expressed in breast cancer cell lines and primary tumor tissues, possibly due to an abnormal trans-differentiation process^51^.

TNBC patients do not respond to hormone-based or anti-HER2 therapies^52^ and treatment options are mostly limited to chemotherapy (neoadjuvant and adjuvant) and surgery. Furthermore, the heterogeneous nature of TNBC limits the efficacy of standard chemotherapeutic agents because the response to these treatments varies greatly among patients. A few targeted therapies for TNBC have been proposed, including mTOR inhibitors, FGFR inhibitors, and EGFR-directed antibodies such as cetuximab^52^. Our results include the discovery that the androgen receptor is expressed in tumors in cluster 2. This finding suggests there may be an opportunity to treat this group of patients with anti-androgens^53^. Also, we identified two oncogenic miRNAs, *miR-10b* and *miR-9*, that are specifically upregulated in clusters 2 and 3, respectively. Silencing of *miR-10b* using an antagomir oligonucleotide^54^ and silencing of *miR-9* using a miRNA sponge^46^ have been shown to inhibit metastasis in mouse models of breast cancer.

Collectively, our results indicate that a new classification system for TNBC that addresses its inherent heterogeneity can be defined by an integrative clustering methodology that incorporates multiple different data types. With this approach, we identified specific molecular signatures for different TNBC clusters. The distinctiveness of the integrative clusters we identified in terms of their genetic and epigenetic properties, oncogenic drivers and clinical features suggests that this new cluster assignment could provide another perspective on TNBC heterogeneity.

## Methods

### Acquisition of patient data

All the sequencing and clinical data associated with TNBC patients (n = 180) were obtained from The Cancer Genome Atlas (TCGA) hosted by the Genomic Data Commons (GDC). Patients (n = 180) were identified as triple negative breast cancer patients based on the analysis performed by Lehmann *et al*.^21^. RNA-Seq and miRNA-Seq data were downloaded as raw counts. The CNV data from Affymetrix SNP 6.0 array was processed by GDC into output files containing segment mean values (transformed copy number values for each of the segmented genomic regions), which were used for this work. DNA methylation levels (as beta values) from Illumina Infinium Human Methylation 450 arrays (if available) were also obtained. (More information on GDC output files and pipelines can be found on the GDC website: https://docs.gdc.cancer.gov/.) Clinical metadata (Supplementary Table S1) were extracted from clinical XML files provided by GDC.

Purity estimates for the 180 TNBC tumors were obtained from Aran *et al*.^55^ and Li *et al*.^30^. Patients were kept in the study if they had a consensus purity estimate (CPE) (from Aran *et al*.) or Clonal Heterogeneity Analysis Tool (CHAT) (from Li *et al*.) purity measurement of 60% or higher. The purity scores are presented in Supplementary Table S1.

### Single data type patient clustering

The workflow for single data type clustering is presented in Fig. 1. The raw reads (genes and miRNAs) were normalized and log_2_ transformed by DESeq2^56^. The segment mean values for CNV events were processed and converted to a CNV region-by-patient matrix using the CNTools bioconductor package^57^. Standard deviation (sd) filters (sd >2, sd >1, and sd >0.5 for gene, miRNA, and CNV, respectively) were used to select the genes, miRNAs, and CNVs that showed maximum variation across the patients and, therefore, were indicative of TNBC inter-tumor heterogeneity. Applying these criteria led to the selection of 2587 genes, 369 miRNAs and 5000 CNVs. We applied consensus clustering using non-negative matrix factorization (NMF) as the underlying algorithm. NMF clustering was performed using the NMF bioconductor package^58^ with Euclidean and Kullback-Leibler divergence as distance measures. The cluster number was selected based on the values of cophentic correlation coefficient^59^, which is a metric for the stability of clusters^58^. We evaluated the cluster solutions from NMF methods by following internal validation metrics^34^: RMSSTD, r-squared and SD validity index.

### Integrative clustering

Integrative clustering using gene, miRNA, and CNV data was performed using the similarity network fusion (SNF) method^36^ for the 134 patients for which all the data were available from TCGA. No preselection of genes, miRNAs, or CNVs was required for SNF method. The three data types were aggregated by SNFtool R package to generate a fused patient network and a fused patient similarity matrix using the following parameters: K = 13, alpha = 0.5, and t = 20^36^. Spectral clustering was performed on the fused patient similarity matrix using the function built into the SNF package. Cluster number was chosen using the supplied function, estimateNumberOfClustersGivenGraph. The networks were visualized in Cytoscape^60^ by generating a *k*-nearest neighbors graph from the values of the patient similarity matrix. Normalized mutual information (NMI) scores of the features (genes, miRNAs, and CNVs) were obtained as the output from SNFtools package using its rankFeaturesByNMI function. For the fused patient network generated using gene, miRNA, CNV, and DNA methylation data, only the 87 of 134 patients with associated methylation data were used.

### Pathway Analysis of genes, miRNAs, and CNVs

Gene ontology (biological process and molecular function) and pathway analysis (based on KEGG^61^ and Reactome^62^ databases of cellular pathways) of genes was performed using g:Cocoa module of g:Profiler^63^. The statistical significance of the results after multiple testing correction was computed using the built-in g:SCS method. Only the results (enriched pathways) with *p* values <0.05 were considered significant. The gene targets for miRNAs were obtained from miRTarBase v6.0^64^ and miRTex^65^. The genes present in the CNV regions were extracted using genomicRanges bioconductor package^66^. SNF cluster-specific data for genomic alterations (mutations and genes amplified or deleted by CNVs) were obtained from cBioPortal for Cancer Genomics^67,68^: http://www.cbioportal.org.

### Differential expression analysis of genes and miRNAs

Differential gene and miRNA expression between the SNF cluster pairs (1 and 2, 2 and 3, and 1 and 3) was performed using DESeq2 bioconductor package^56^. Only the significant (adjusted p < 0.05) genes and miRNAs with absolute log_2_fold change >1 were selected. In order to find cluster-specific genes, we compared the log_2_fold-change values of the genes across the three pairwise differential expression analyses. Genes were considered specifically upregulated (downregulated) in one cluster if they were upregulated (downregulated) in that cluster compared to each of the other two clusters.

### Predictive modeling

Classifiers were created using the caret package in R^69^. Initial predictors were selected by taking the top-ranked NMI features from the SNF network analysis (top 1% for genes and CNVs and top 5% for miRNA). A 70/30 split was applied to the data (n = 134) to create a training set (n = 94) and a test set (n = 40). Samples were selected for the training set by the partition function built into the caret software package. Predictor selection was performed on the training set by applying a Kruskal-Wallis test across the top NMI features (FDR < 0.01). Predictors that passed the significance test (525 genes, 112 miRNAs, and 1809 CNVs) were used for the classifiers. Three classifiers (supplied by the carat package) were trained: elastic net logistic regression (glmnet function), random forest (randomForest), and a linear kernel support vector machine (lmSVM). Leave-one-out cross validation (LOOCV) was used to further validate and tune model parameters and the resulting classifiers were evaluated using accuracy and average F1 scores (based on precision and recall) obtained from the carat package (Supplementary Table S7).

### Statistical analysis

Statistical tests employed for each analysis are provided in the text and legends. For all analyses, we used adjusted p < 0.05 to determine statistical significance. p values are adjusted for multiple hypothesis testing. Tests are two-sided.

### Data availability

All the datasets used in the study are publicly available from NCI genomic data commons: https://gdc.cancer.gov/.

### Code availability

The software packages used in this study are listed in Supplementary Table S8. Further information is available upon request.

## Electronic supplementary material

Supplementary Information

Table S1

Table S2

Table S3

Table S4

Table S5

Table S6

Table S7

Table S8

## References

[1] Polyak K (2011). Heterogeneity in breast cancer. J Clin Invest.

[2] Koren S, Bentires-Alj M (2015). Breast Tumor Heterogeneity: Source of Fitness, Hurdle for Therapy. Mol Cell.

[3] Zardavas D, Irrthum A, Swanton C, Piccart M (2015). Clinical management of breast cancer heterogeneity. Nat Rev Clin Oncol.

[4] Prat A, Perou CM (2011). Deconstructing the molecular portraits of breast cancer. Mol Oncol.

[5] Perou CM (2000). Molecular portraits of human breast tumours. Nature.

[6] Prat A (2015). Clinical implications of the intrinsic molecular subtypes of breast cancer. Breast.

[7] Prat A (2010). Phenotypic and molecular characterization of the claudin-low intrinsic subtype of breast cancer. Breast Cancer Res.

[8] Cancer Genome Atlas N (2012). Comprehensive molecular portraits of human breast tumours. Nature.

[9] Blows FM (2010). Subtyping of breast cancer by immunohistochemistry to investigate a relationship between subtype and short and long term survival: a collaborative analysis of data for 10,159 cases from 12 studies. Plos Med.

[10] Livasy CA (2006). Phenotypic evaluation of the basal-like subtype of invasive breast carcinoma. Mod Pathol.

[11] Xu H, Eirew P, Mullaly SC, Aparicio S (2014). The omics of triple-negative breast cancers. Clin Chem.

[12] Bauer KR, Brown M, Cress RD, Parise CA, Caggiano V (2007). Descriptive analysis of estrogen receptor (ER)-negative, progesterone receptor (PR)-negative, and HER2-negative invasive breast cancer, the so-called triple-negative phenotype: a population-based study from the California cancer Registry. Cancer.

[13] Dent R (2007). Triple-negative breast cancer: clinical features and patterns of recurrence. Clin Cancer Res.

[14] Metzger-Filho O (2012). Dissecting the heterogeneity of triple-negative breast cancer. J Clin Oncol.

[15] Bianchini G, Balko JM, Mayer IA, Sanders ME, Gianni L (2016). Triple-negative breast cancer: challenges and opportunities of a heterogeneous disease. Nat Rev Clin Oncol.

[16] Pareja F (2016). Triple-negative breast cancer: the importance of molecular and histologic subtyping, and recognition of low-grade variants. NPJ Breast Cancer.

[17] Shah SP (2012). The clonal and mutational evolution spectrum of primary triple-negative breast cancers. Nature.

[18] Lehmann BD (2011). Identification of human triple-negative breast cancer subtypes and preclinical models for selection of targeted therapies. J Clin Invest.

[19] Prat A (2013). Molecular characterization of basal-like and non-basal-like triple-negative breast cancer. Oncologist.

[20] Burstein MD (2015). Comprehensive genomic analysis identifies novel subtypes and targets of triple-negative breast cancer. Clin Cancer Res.

[21] Lehmann BD (2016). Refinement of Triple-Negative Breast Cancer Molecular Subtypes: Implications for Neoadjuvant Chemotherapy Selection. Plos One.

[22] Stirzaker C (2015). Methylome sequencing in triple-negative breast cancer reveals distinct methylation clusters with prognostic value. Nat Commun.

[23] Kristensen VN (2014). Principles and methods of integrative genomic analyses in cancer. Nat Rev Cancer.

[24] Wang D, Gu J (2016). Integrative clustering methods of multi-omics data for molecule-based cancer classifications. Quantitative Biology.

[25] Curtis C (2012). The genomic and transcriptomic architecture of 2,000 breast tumours reveals novel subgroups. Nature.

[26] Kristensen VN (2012). Integrated molecular profiles of invasive breast tumors and ductal carcinoma *in situ* (DCIS) reveal differential vascular and interleukin signaling. Proc Natl Acad Sci USA.

[27] Cavalli FMG (2017). Intertumoral Heterogeneity within Medulloblastoma Subgroups. Cancer Cell.

[28] TCGA Tissue Sample Requirements: High Quality Requirements Yield High Quality Data. Available at, https://cancergenome.nih.gov/cancersselected/biospeccriteria (Accessed on November 2016).

[29] Aran D, Butte AJ (2016). Digitally deconvolving the tumor microenvironment. Genome Biol.

[30] Li B, Li JZ (2014). A general framework for analyzing tumor subclonality using SNP array and DNA sequencing data. Genome Biol.

[31] Li B (2016). Comprehensive analyses of tumor immunity: implications for cancer immunotherapy. Genome Biol.

[32] Monti S, Tamayo P, Mesirov J, Golub T (2003). Consensus Clustering: A Resampling-Based Method for Class Discovery and Visualization of Gene Expression Microarray Data. Machine Learning.

[33] Devarajan K (2008). Nonnegative matrix factorization: an analytical and interpretive tool in computational biology. Plos Comput Biol.

[34] Ronan T, Qi Z, Naegle KM (2016). Avoiding common pitfalls when clustering biological data. Sci Signal.

[35] Wagner, S. & Wagner, D. Comparing clusterings: an overview. *Univ*, *Fak für Informatik*, 2007 (2007).

[36] Wang B (2014). Similarity network fusion for aggregating data types on a genomic scale. Nat Methods.

[37] Emmons S, Kobourov S, Gallant M, Borner K (2016). Analysis of Network Clustering Algorithms and Cluster Quality Metrics at Scale. Plos One.

[38] Amin, M. B. *et al*. *AJCC Cancer Staging Manual*, 8 edn. Springer International Publishing (2017).

[39] Rampurwala M, Wisinski KB, O’Regan R (2016). Role of the androgen receptor in triple-negative breast cancer. Clin Adv Hematol Oncol.

[40] Ma L, Teruya-Feldstein J, Weinberg RA (2007). Tumour invasion and metastasis initiated by microRNA-10b in breast cancer. Nature.

[41] Shenouda SK, Alahari SK (2009). MicroRNA function in cancer: oncogene or a tumor suppressor?. Cancer Metastasis Rev.

[42] Wang W, Luo YP (2015). MicroRNAs in breast cancer: oncogene and tumor suppressors with clinical potential. J Zhejiang Univ Sci B.

[43] Hossain A, Kuo MT, Saunders GF (2006). Mir-17-5p regulates breast cancer cell proliferation by inhibiting translation of AIB1 mRNA. Mol Cell Biol.

[44] Keklikoglou I (2015). MicroRNA-206 functions as a pleiotropic modulator of cell proliferation, invasion and lymphangiogenesis in pancreatic adenocarcinoma by targeting ANXA2 and KRAS genes. Oncogene.

[45] Xiao H (2016). miR-206 functions as a novel cell cycle regulator and tumor suppressor in clear-cell renal cell carcinoma. Cancer Lett.

[46] Ma L (2010). miR-9, a MYC/MYCN-activated microRNA, regulates E-cadherin and cancer metastasis. Nat Cell Biol.

[47] Vogelstein B (2013). Cancer genome landscapes. Science.

[48] Shlien A, Malkin D (2009). Copy number variations and cancer. Genome Med.

[49] Lehmann BD (2014). PIK3CA mutations in androgen receptor-positive triple negative breast cancer confer sensitivity to the combination of PI3K and androgen receptor inhibitors. Breast Cancer Res.

[50] Skibinski A, Kuperwasser C (2015). The origin of breast tumor heterogeneity. Oncogene.

[51] Zhang Q, Fan H, Shen J, Hoffman RM, Xing HR (2010). Human breast cancer cell lines co-express neuronal, epithelial, and melanocytic differentiation markers *in vitro* and *in vivo*. Plos One.

[52] Wahba HA, El-Hadaad HA (2015). Current approaches in treatment of triple-negative breast cancer. Cancer Biol Med.

[53] Barton VN, Gordon MA, Richer JK, Elias A (2016). Anti-androgen therapy in triple-negative breast cancer. Ther Adv Med Oncol.

[54] Ma L (2010). Therapeutic silencing of miR-10b inhibits metastasis in a mouse mammary tumor model. Nat Biotechnol.

[55] Aran D, Sirota M, Butte AJ (2015). Systematic pan-cancer analysis of tumour purity. Nat Commun.

[56] Love MI, Huber W, Anders S (2014). Moderated estimation of fold change and dispersion for RNA-seq data with DESeq2. Genome Biol.

[57] Zhang, J. CNTools: Convert segment data into a region by sample matrix to allow for other high level computational analyses. R package version 1.34.0 (2017).

[58] Gaujoux R, Seoighe C (2010). A flexible R package for nonnegative matrix factorization. BMC Bioinformatics.

[59] Brunet JP, Tamayo P, Golub TR, Mesirov JP (2004). Metagenes and molecular pattern discovery using matrix factorization. Proc Natl Acad Sci USA.

[60] Cline MS (2007). Integration of biological networks and gene expression data using Cytoscape. Nat Protoc.

[61] Kanehisa M, Goto S (2000). KEGG: kyoto encyclopedia of genes and genomes. Nucleic Acids Res.

[62] Joshi-Tope G (2005). Reactome: a knowledgebase of biological pathways. Nucleic Acids Res.

[63] Reimand J, Kull M, Peterson H, Hansen J, Vilo J (2007). g:Profiler–a web-based toolset for functional profiling of gene lists from large-scale experiments. Nucleic Acids Res.

[64] Hsu SD (2011). miRTarBase: a database curates experimentally validated microRNA-target interactions. Nucleic Acids Res.

[65] Li G (2015). miRTex: A Text Mining System for miRNA-Gene Relation Extraction. Plos Comput Biol.

[66] Lawrence M (2013). Software for computing and annotating genomic ranges. Plos Comput Biol.

[67] Cerami E (2012). The cBio cancer genomics portal: an open platform for exploring multidimensional cancer genomics data. Cancer Discov.

[68] Gao J (2013). Integrative analysis of complex cancer genomics and clinical profiles using the cBioPortal. Sci Signal.

[69] Kuhn, M. Building predictive models in R using the caret package. *Journal of Statistical Software* **28** (2008).

[70] Lex A (2012). StratomeX: Visual Analysis of Large-Scale Heterogeneous Genomics Data for Cancer Subtype Characterization. Comput Graph Forum.

