# Supplementary material for: Integrative analysis of the inter-tumoral heterogeneity of triple-negative breast cancer
Source: Sci Rep. 2018 Aug 7;8:11807. doi: 10.1038/s41598-018-29992-5 (PMC6081411; doi:10.1038/s41598-018-29992-5)

Supplementary Information

Integrative analysis of the inter-tumoral heterogeneity of triple-negative breast cancer

Alec M. Chiu[1], Mithun Mitra[2,3], Lari Boymoushakian[4], and Hilary A. Coller[1,2,3*]

[1]Bioinformatics Interdepartmental Program, University of California, Los Angeles

[2]Department of Molecular, Cell, and Developmental Biology, University of California, Los Angeles

[3]Department of Biological Chemistry, David Geffen School of Medicine, University of California, Los Angeles

[4]Department of Computer Science, University of California, Los Angeles

*Correspondence and request for materials should be addressed to H.A.C. (hcoller@ucla.edu)

Supplementary Figures

Figure S1: Heatmap representation of patient similarity matrices for single-data clustering. Gene-based (A), miRNA-based (B), and CNV-based (C) clustering was performed using NMF method. The patients in the heatmaps are grouped based on the cluster labels. Only the best clustering solutions based on validation metrics are shown. The white regions represent low patient-to-patient similarity, while darker colors indicate regions with high similarity between the patients.

Figure S2: Classification of single-data NMF clusters based on PAM50 and Lehmann subtypes.

Figure S3: SNF integrative network obtained using gene, miRNA, CNV, and methylation data for a subset (n=87) of patients used for SNF (n=134) clustering. For this subset of patient, all the data including the methylation data from Illumina Infinium Human Methylation 450 array iare available from TCGA. The patients in the network are color-coded based on their assignment to the three SNF clusters.

Figure S4: Classification of SNF clusters based on single-data clusters.

Figure S5: Phenogram showing top 1% of NMI-scored CNVs based on SNF network analysis. Also shown are the genes associated with CNV regions.

Figure S6: Variation of up_2 and down_2 genes (A and B) and up_2 and down_2 miRNAs (C and D) across the three SNF clusters. The SNF clusters are further classified into PAM50 (A and C) or Lehmann subtypes (B and D).

Figure S1

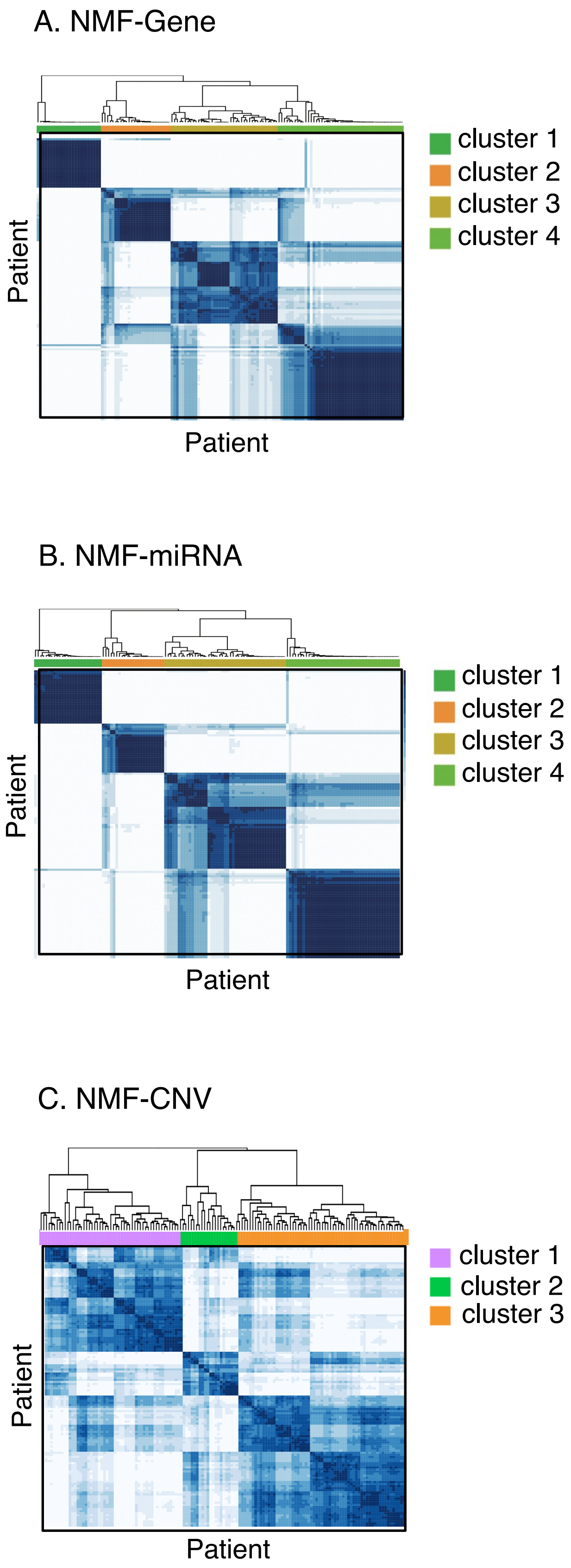
A. NMF-Gene
Patient
Patient
cluster 1
cluster 2
cluster 3
cluster 4
B. NMF-miRNA
Patient
Patient
cluster 1
cluster 2
cluster 3
cluster 4
C. NMF-CNV
Patient
Patient
cluster 1
cluster 2
cluster 3

Figure S2

A. NMF-gene

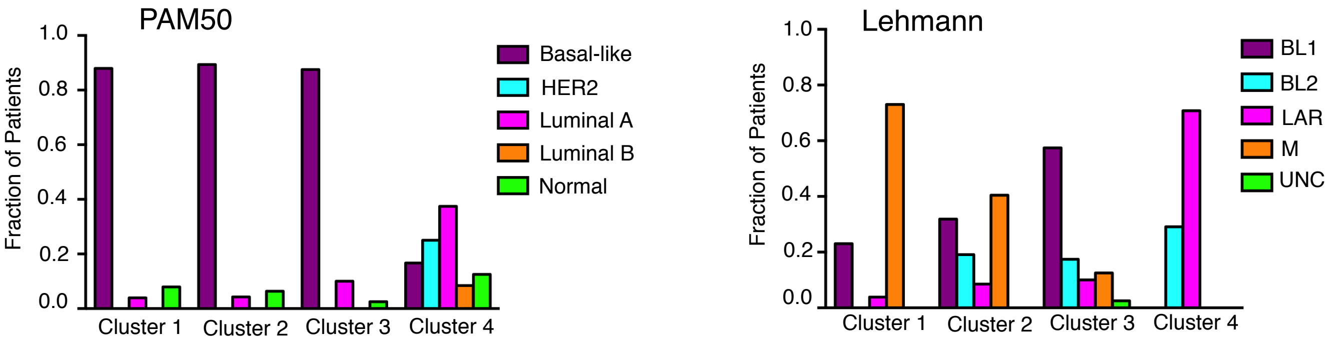

B. NMF-miRNA

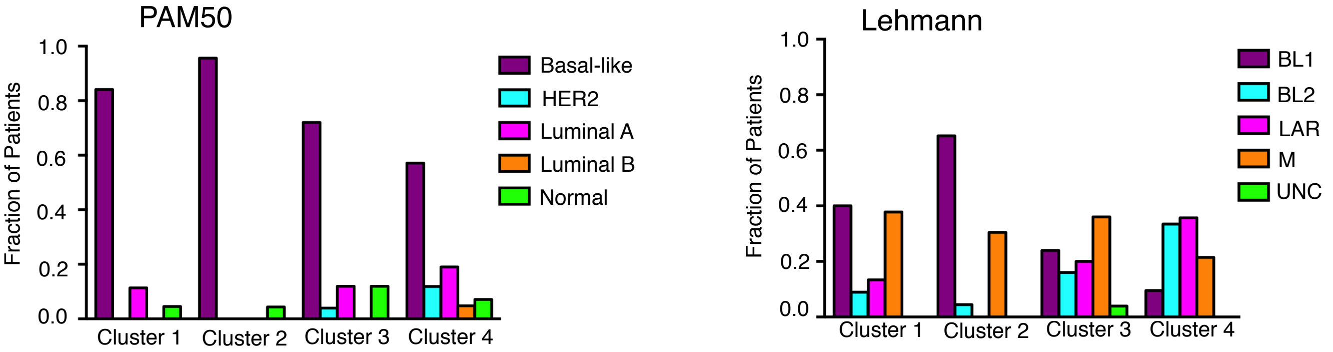

C. NMF-CNV

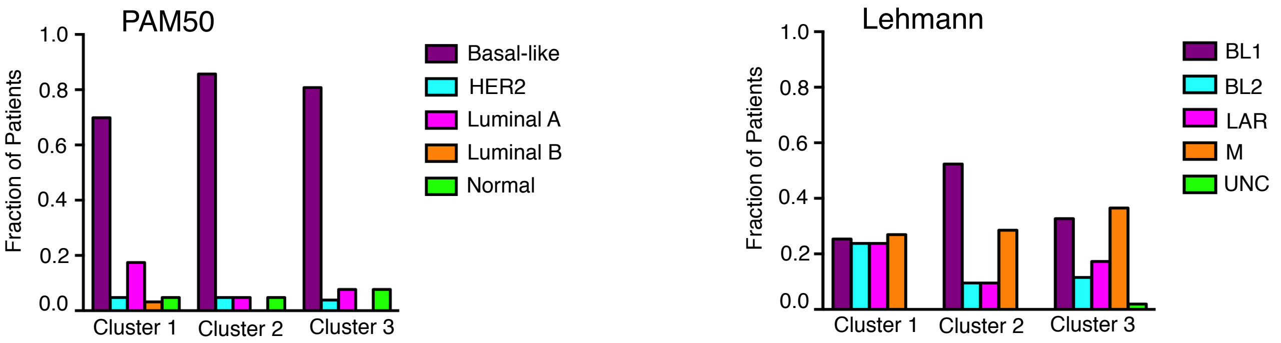

Figure S3

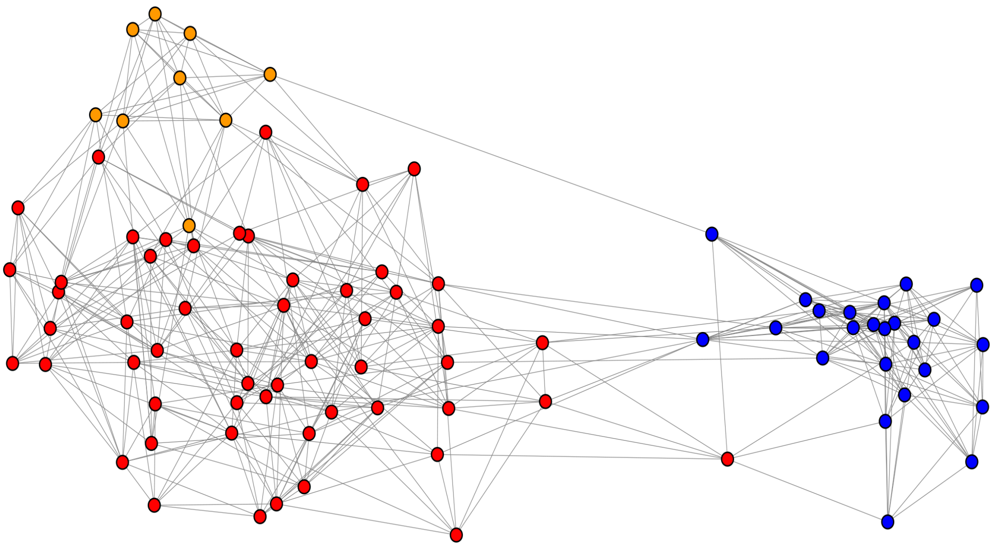

Figure S4

A. SNF clusters vs. NMF-gene clusters

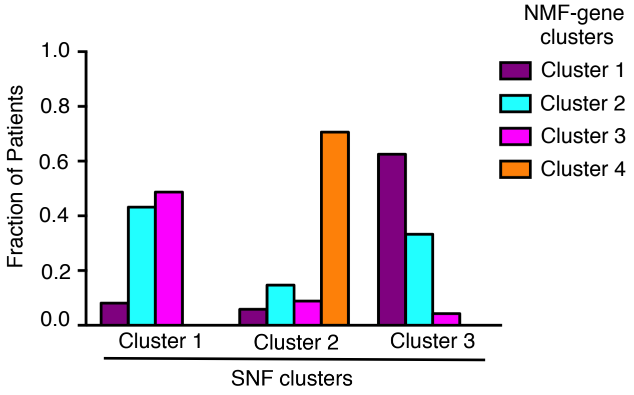

B. SNF clusters vs. NMF-miRNA clusters

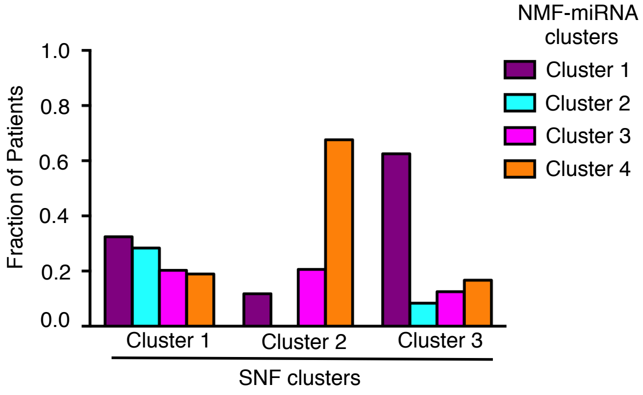

C. SNF clusters vs. NMF-CNV clusters

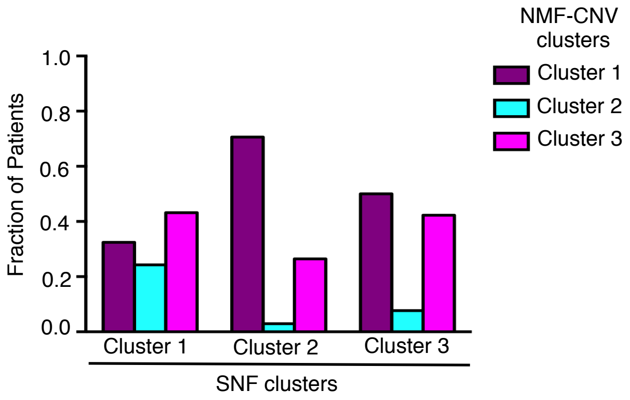

Figure S5

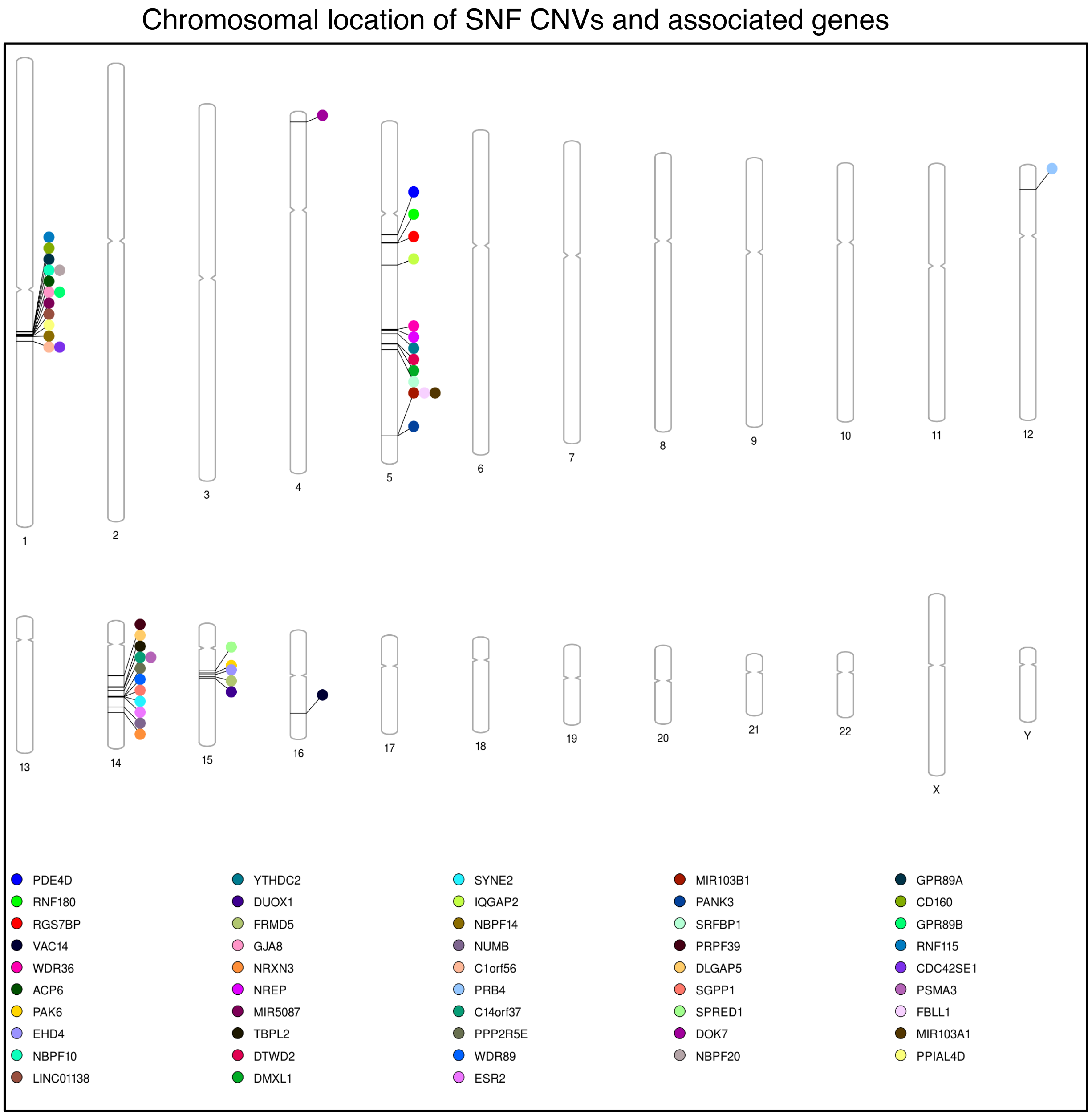
Chromosomal location of SNF CNVs and associated genes
1
2
3
4
5
6
7
8
9
10
11
12
13
14
15
16
17
18
19
20
21
22
X
Y
PDE4D
RNF180
RGS7BP
VAC14
WDR36
ACP6
PAK6
EHD4
NBPF10
LINC01138
YTHDC2
DUOX1
FRMD5
GJA8
NRXN3
NREP
MIR5087
TBPL2
DTWD2
DMXL1
SYNE2
IQGAP2
NBPF14
NUMB
C1orf56
PRB4
C14orf37
PPP2R5E
WDR89
ESR2
MIR103B1
PANK3
SRFBP1
PRPF39
DLGAP5
SGPP1
SPRED1
DOK7
NBPF20
GPR89A
CD160
GPR89B
RNF115
CDC42SE1
PSMA3
FBLL1
MIR103A1
PPIAL4D

Figure S6

A. up_2 and down_2 genes

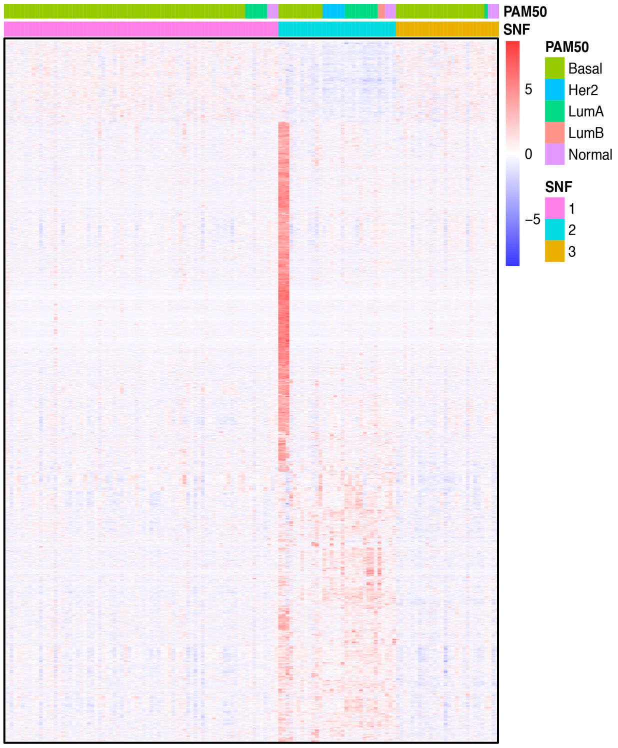

B. up_2 and down_2 genes

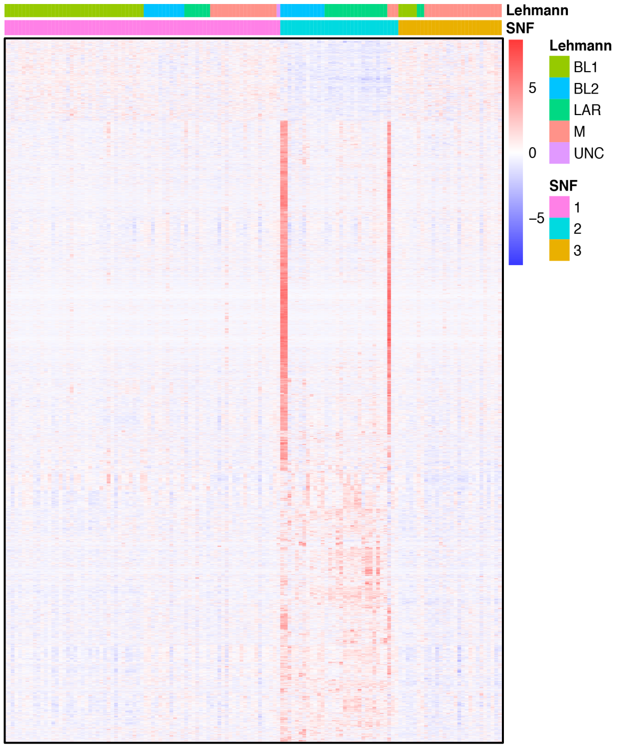

C. up_2 and down_2 miRNAs

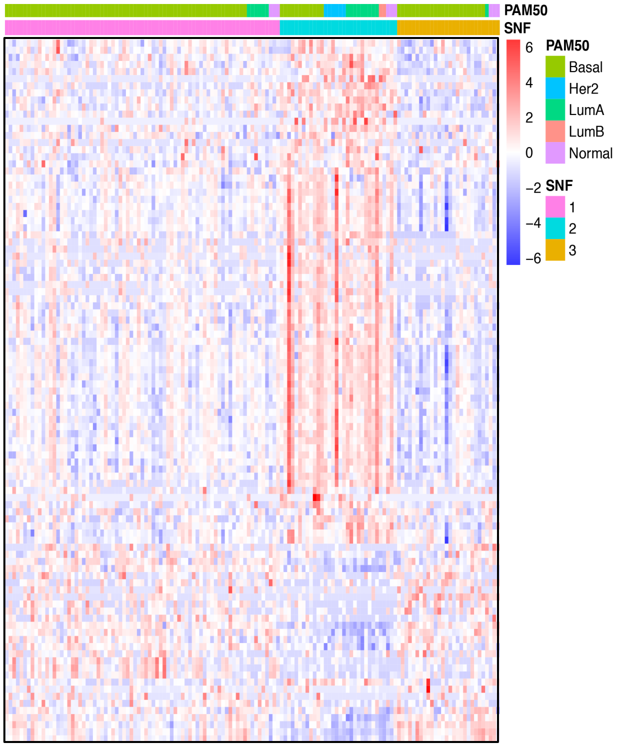

D. up_2 and down_2 miRNAs

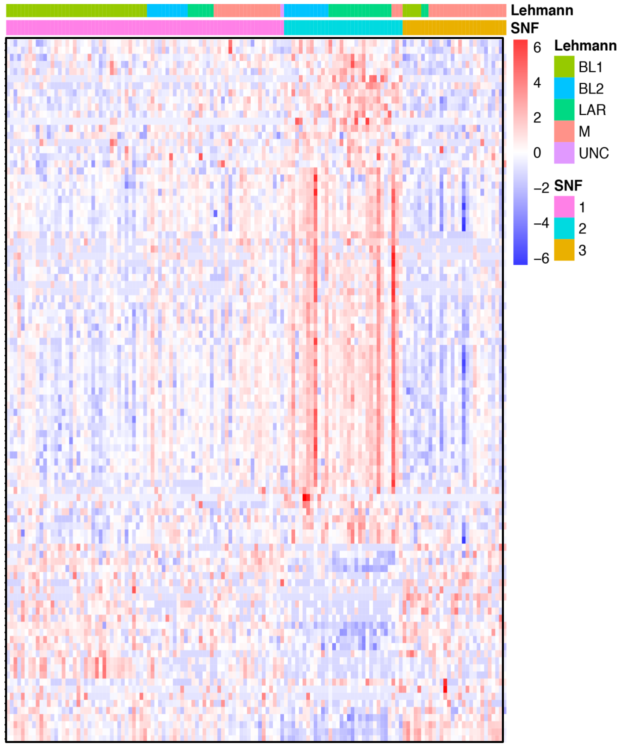

Supplement: Supplementary file 1 — Supplementary Information [file 41598_2018_29992_MOESM1_ESM.pdf]
